# Genomic Characterization of the Probiotic *Limosilactobacillus mucosae* CRL2069 Isolated from Feedlot Cattle Feces

**DOI:** 10.3390/microorganisms14102270

**Published:** 2026-10-06

**Authors:** Cecilia M. Aristimuño Ficoseco, Natalia Carrasco, Marco Montemurro, Daniele Chieffi, Silvina G. Fadda, Maria E. F. Nader-Macias, Vincenzina Fusco, Graciela M. Vignolo, Francesca Fanelli

**Affiliations:** 1 Reference Center for Lactobacilli (CERELA-CONICET), San Miguel de Tucumán 4000, Argentina; 2 Institute of Sciences of Food Production (CNR-ISPA), National Research Council, 70126 Bari, Italy

**Keywords:** *Limosilactobacillus mucosae*, genomics, probiotics, stress, CAZymes, safety assessment

## Abstract

*Limosilactobacillus mucosae* (L.) CRL2069, isolated from feedlot cattle feces, has previously exhibited high performance when used as a probiotic in animals. To investigate the genetic bases underlying its probiotic potential, complete genome sequencing and comparative analyses were performed. The CRL2069 genome showed a total length of 1.93 Mb, with an average GC content of 46.76%. No evidence of plasmid sequences was detected, while CRISPR-Cas elements, as well as twelve genomic islands, were identified in the genome. Pan-genomic and clustering analysis among *L. mucosae* strains showed a lack of partitioning according to their isolation origin, revealing a niche specialization. CRL2069 genes related to stress and those conferring probiotic character were identified. In addition, the absence of acquired antimicrobial resistance genes and virulence determinants evidenced the safety of this strain. Genomic determinants conferring the ability to metabolize a diverse array of carbohydrates, particularly those present in cattle feed, were annotated. Phenotypic analysis supported the genomic prediction and demonstrated the capacity of CRL2069 to metabolize 39 out of 95 carbon sources tested and to produce folate. Based on the results obtained, *L. mucosae* CRL2069 evaluated in this study is confirmed to be a promising candidate for use as a probiotic in cattle.

## 1. Introduction

Intensive food animal rearing and/or fattening systems (feedlots) may compromise animal welfare, often leading to stress and health issues. The main goal of these practices is to maximize growth performance and animal productivity while maintaining meat quality [1]. During the last century, antibiotics were routinely used as growth promoters in animal feed. This practice has contributed to the global crisis of antimicrobial resistance by the emergence of resistant bacteria, compromising the effectiveness of antimicrobial drugs for treating human infections [2]. The European Union and the FDA [3,4] banned the use of antibiotics as animal growth promoters in 2006 and 2017, respectively. These regulatory changes have constituted a driving force for the search for safer alternatives, such as probiotics. In food animal production, these beneficial microbes have been shown to optimize feed utilization and support growth and productivity, thereby contributing to more sustainable farming practices; beyond these roles, probiotics also mitigate antimicrobial resistance by offering natural alternatives to conventional treatments [5].

The Food and Agriculture Organisation and the World Health Organisation (FAO/WHO) define probiotics as “live microorganisms that, when ingested in adequate amounts, confer a health benefit on the host” [6,7,8]. Lactic acid bacteria (LAB) are the most widely used commercial probiotics for improving animal and human health. LAB are found in the gastrointestinal tract (GIT) of humans and animals, playing a key physiological role in maintaining intestinal homeostasis [9]. In the bovine host, treatments with probiotic microorganisms were reported to enhance gut health, modulate the immune system, and suppress pathogens, leading to improved growth performance and disease prevention [5,10]. The production of antimicrobial compounds, the inhibition of pathogen adhesion/aggregation, the modulation of the intestinal microbiota and the improvement of immune responses were identified as mechanisms of probiotic action in cattle hosts [5,10,11].

Although probiotic strains include other genera such as *Bifidobacterium*, *Pediococcus*, *Lactococcus*, and *Enterococcus*, most probiotics belong to the genus formerly known as *Lactobacillus*, a taxonomically heterogeneous group reclassified into 25 genera including more than 170 species [12]. The name of the genus *Limosilactobacillus* refers to the ability of most strains to produce exopolysaccharides exhibiting a slimy characteristic (from the Latin *limosus*). Within this genus, the species *Limosilactobacillus mucosae* includes obligate heterofermentative strains, which have been isolated from intestinal habitats of vertebrates including animals [13,14,15,16,17,18] and humans [19,20], as well as from cereal fermentations [21]. Many strains of this species carry a mucus-binding protein as a putative colonization factor; this adhesion protein is required for the bacteria to survive in an open-flow environment such as the GIT. The ability of *L. mucosae* to colonize the intestinal mucus layer, together with its occurrence in other mucosal niches such as the human cervix and vagina, highlights its strong affinity for mucins and host mucosal surfaces [22].

The European Food Safety Authority (EFSA) has published guidelines for the safety assessment of probiotics, including accurate taxonomic identification of the strain based on whole-genome sequencing (WGS) and genomic and phenotypic determination of antibiotic resistance profile and virulence factors [23]. This preliminary evaluation of the probiotic potential of a newly isolated strain is thus essential before proceeding to the assessment of its health benefits and the incorporation into food, veterinary, or pharmaceutical formulations [24].

In a previous study by our group, the identification and diversity of the LAB population associated with feedlot cattle feces were reported [11,15]. Among the identified lactobacilli, *L. mucosae* CRL2069 has been shown to resist gastrointestinal conditions, to lack antibiotic resistance and virulence factors, to enhance nutritional and metabolic balance, to modulate Toll-like receptor-mediated innate immunity in bovine intestinal epithelial cells and to maintain viability and stability after the freeze-drying and storage processes [11,15,25,26,27]. The aim of this study was to perform a comprehensive genomic and phenotypic characterization of *L. mucosae* CRL2069 to elucidate the genetic basis of its probiotic potential. To this end, a genomic analysis was conducted to identify genes involved in the metabolism and transport of various compounds, adherence to bovine intestinal epithelial cells, exopolysaccharide production, and virulence or antibiotic resistance factors. In addition, sugar consumption and vitamin B production were phenotypically evaluated. Overall, the combined genomic and phenotypic evidence provides novel insights and further supports the safety, functional potential and suitability of this strain as a probiotic candidate for livestock production.

## 2. Material and Methods

### 2.1. Strain Info and Culture Conditions

*L. mucosae* CRL2069 was stored at −80 °C in milk–yeast extract (13% non-fat milk, 0.5% yeast extract, and 1% glucose) with 20% glycerol. The inoculum of the strain was prepared by transferring stock cultures to MRS broth (Merck, Darmstadt, Germany) and sub-culturing twice in the same medium at 37 °C for 16 h.

### 2.2. Whole-Genome Sequencing

Genomic DNA was extracted following the method outlined by Pospiech and Neumann [28]. DNA integrity, purity, and concentration were evaluated using agarose gel electrophoresis, a Nanodrop spectrophotometer (Peqlab Biotechnologie GmbH, Erlangen, Germany), and a Qubit 3.0 fluorometer (Life Technologies, Carlsbad, CA, USA). Whole-genome shotgun sequencing was carried out by Novogene Bioinformatics Technology Co., Ltd. (Beijing, China) using the Illumina NovaSeq platform (San Diego, CA, USA), with the 2 × 150 bp mate-pair protocol. Sequencing reads were processed with NxTrim (v2) [29], de novo genome assembly was conducted using SKESA [30] and integrated into the read assembly and annotation pipeline tool (RAPT; https://github.com/ncbi/rapt/; accessed on 1 September 2024). The whole-genome shotgun project has been deposited at DDBJ/ENA/GenBank under the accession JAXKWS000000000. The version described in this paper is JAXKWS010000000.

### 2.3. Bioinformatic Methods

#### 2.3.1. Genome Assembly and Quality Assessment

The overall contiguity and assembly statistics were assessed using Quast (v5.3.0; https://github.com/ablab/quast; accessed on 20 September 2024), while contamination and completeness were evaluated by using CheckM2 (v1.1.0; https://github.com/chklovski/CheckM2; accessed on 20 September 2024). Assembly completeness was further evaluated with Busco (v6.6.0; https://gitlab.com/ezlab/busco; accessed on 21 September 2024) using the *bacteria_odb10* lineage dataset and analyzing the presence of 124 universally conserved single-copy genes in nearly all prokaryotic genomes.

#### 2.3.2. Genome Annotation and Phylogenomic and Comparative Analysis

Gene prediction for *L. mucosae* CRL2069 was performed using a multi-tool approach: the NCBI Prokaryotic Genome Annotation Pipeline (PGAP; [31]) the Comprehensive Genome Analysis Service available through the Bacterial and Viral Bioinformatics Resource Center (BV-BRC; https://www.bv-brc.org/; accessed on 10 October 2024), and the Prokka annotation pipeline [32]. The resulting annotations were subsequently refined through manual curation. Protein identifiers referenced throughout the manuscript correspond to those generated by PGAP.

Phylogenetic analysis was performed employing the Bacterial Genome Tree service implemented in the BV-BRC (version 3.35.5; https://www.bv-brc.org/; accessed on 2 February 2025) using the maximum likelihood method implemented in RAxML (version 8.2.11) with progressive refinement [33]. For this study, representative genomes from all 33 officially recognized species within the *Limosilactobacillus* genus (Supplementary Table S1) were analyzed. The BV-BRC pipeline identified 134 conserved single-copy genes shared among the selected genomes, which were used as marker genes for phylogenetic reconstruction. *Paucilactobacillus oligofermentans* DSM 15707 was included as an outgroup. Phylogenetic trees were visualized using iTOL (version 6.9; [34]).

Comparative analysis was performed on 54 genomes publicly available at the time of this study and filtered by quality, excluding WGS obtained from assembled shotgun metagenomic data, as indicated in the NCBI genomes dataset (https://www.ncbi.nlm.nih.gov/datasets/genome/?taxon=97478; accessed on 1 March 2025). Pangenome reconstruction was performed using Roary (v3.12.0; [35]), with default options. Core genome phylogeny was inferred using IQ-TREE [36] with the GTR+G substitution model. Branch support was assessed using ultrafast bootstrap (1000 replicates) and the approximate likelihood ratio test (SH-aLRT, 1000 replicates), with automatic thread allocation.

The core genome was functionally annotated for Cluster of Orthologous Groups (COG) categories using eggNOG-mapper (version 2.1.13; [37]).

### 2.4. In Silico Evaluation of Probiotic Potential

The Basic Local Alignment Search Tool (BLAST) algorithms were used to perform homology-based analysis toward reference sequences (http://blast.ncbi.nlm.nih.gov/Blast.cgi; accessed on 2 April 2025). The evaluation of the probiotic potential of *L. mucosae* CRL2069 was performed by homology-based analysis with reference proteins associated with probiotic features in the *Limosilactobacillus* genus [38,39,40]. Homology was predicted by BLASTP with a cutoff E-value of 1 × 10^−10^ and minimum identity of 35% and then manually curated.

Carbohydrate-active enzymes (CAZymes) were identified by executing the dbCAN annotation tool (version 4.1.1; [41]). Functional assessments were manually verified using the InterPro [42] and UniProt [43] databases. CAZymes clustering was performed using the average linkage method based on the Euclidean distances.

Putative gene clusters involved in the biosynthesis of ribosomally synthesized and post-translationally modified peptides (RiPPs) and (unmodified) bacteriocins were predicted using antiSMASH (version 7.0; [44]). The PHASTEST webserver (PHAge Search Tool with Enhanced SequenceTranslation; [45]) was used to identify prophage sequences within the bacterial genome. Genomic islands (GIs) were predicted by using the IslandViewer webserver [46]. Contigs were rearranged using *L. mucosae* strain LM1 as the reference genome. The CRISPRCasFinder webserver [47] was used to detect CRISPR direct repeats and spacers.

The Resistance Gene Identifier (RGI) tool implemented in the Comprehensive Antibiotic Resistance Database (CARD; https://card.mcmaster.ca/; accessed on 15 September 2025) was used to predict the *L. mucosae* CRL2069 resistome. In addition, a *k-mer*-based detection method for antimicrobial resistance genes was performed by using the Comprehensive Genome Analysis Service of BV-BRC. Proteins associated with antimicrobial resistance were also retrieved by keyword search within the UniProt ID entry list obtained by functional annotation. Unless specified, tools were used with default parameters.

### 2.5. EPS Production

*L. mucosae* CRL2069, *Leuconostoc pseudomesenteroides* 20193^T^ (positive control), and *Weissella diestrammenae* DSM27940^T^, previously characterized as a non-EPS-producing strain (negative control; [48]), were grown (10 µL of 10^9^ CFU/mL broth culture inoculated by spread plating) on agar plates containing modified de Man, Rogosa, and Sharpe medium (MRS, Oxoid, Milan, Italy) supplemented with sterile sugar (purity > 99.5%, Sigma, Merck, Darmstadt, Germany) solutions to obtain a final concentration of 20 g/L of either glucose, sucrose, fructose, or galactose. All strains were cultivated for 24 h as previously described and inoculated by streaking in triplicate on MRS–sugar agar media. After incubation at 37 °C for 48 and 72 h, the strains that produced mucoid (slimy) colonies were recorded as capable of producing EPS.

### 2.6. Carbon Source Utilization

The pattern of carbon source utilization by *L. mucosae* CRL2069 was determined using the Biolog AN MicroPlates system (Biolog Inc., Hayward, CA, USA), which contained 95 different carbon sources. Briefly, the strain was grown in MRS broth (Oxoid, Milan, Italy) for 24 h. Cells were then collected by centrifugation (10,000× *g*, for 10 min), washed twice with sterile phosphate buffer (50 mmol/L, pH 7.0) and then resuspended in sterile physiological saline solution (0.9 *w*/*v* NaCl). Each well was inoculated with 100 μL of the bacterial suspension adjusted to 65% transmittance and subsequently incubated at 37 °C for 24 h under anaerobic conditions as recommended by the manufacturer. Positive reactions were automatically recorded using the MicroStation microplate reader (Biolog Inc., Hayward, CA, USA) at 590 nm and 750 nm.

### 2.7. Qualitative Determination of B-Group Vitamins Production

The ability of *L. mucosae* CRL2069 to produce B-group vitamins (folates and riboflavin) was assessed by successive cultivations in selective media: Folic Acid Casei Medium (Difco, Becton Dickinson, Franklin Lakes, NJ, USA) for folate determination and Riboflavin Assay Medium (Becton Dickinson, Franklin Lakes, NJ, USA) for riboflavin determination, as described by Laiño et al. [49] and del Valle et al. [50].

## 3. Results and Discussion

### 3.1. General Features of L. mucosae CRL2069 Genome

*Limosilactobacillus mucosae* CRL2069 (*L. mucosae*) is a strain isolated from the feces of steers raised in intensive fattening systems and previously selected for its probiotic-associated traits. These include cell surface properties, production of antimicrobial compounds against *Listeria monocytogenes*, hydrogen peroxide production, amylolytic activity, biofilm formation, tolerance to GIT conditions, absence of antibiotic resistance genes and virulence factors, and its ability to grow to high cell densities in low-cost culture media. In addition, this strain has been proposed as an immunobiotic candidate for protecting the bovine host from TLR-mediated intestinal inflammatory damage [11,15,25,26,27,51,52,53]. To further investigate the genetic basis underlying these characteristics, the genome of *L. mucosae* CRL2069 was sequenced and analyzed. The general genomic features of *L. mucosae* CRL2069 are shown in Table 1.

The assembled genome has a total length of 1.93 Mb and an N50 of 301,383 bp, with an average GC content of 46.76%. No plasmid sequences were detected: screening with PlasmidFinder did not identify known plasmid replicons, and the assembled contigs showed coverage patterns consistent with chromosomal origin. The final assembly quality was excellent. The genome size and GC content of CRL2069 fell within the genomic diversity previously reported for *L. mucosae* strains (genome size: 1.93–2.40 Mb; GC content: 45.96–48.59%) isolated from different ecological niches, including cattle, pigs, poultry, humans and wild boars [54,55,56,57,58,59]. As reported for other *L. mucosae* strains, GC content was found to be higher than the average GC (40.70%) reported for species of the *Lactobacillus* genus, generally considered a low-GC% genus [60].

#### 3.1.1. Functional Annotation and Metabolic Potential

**Figure 1:**
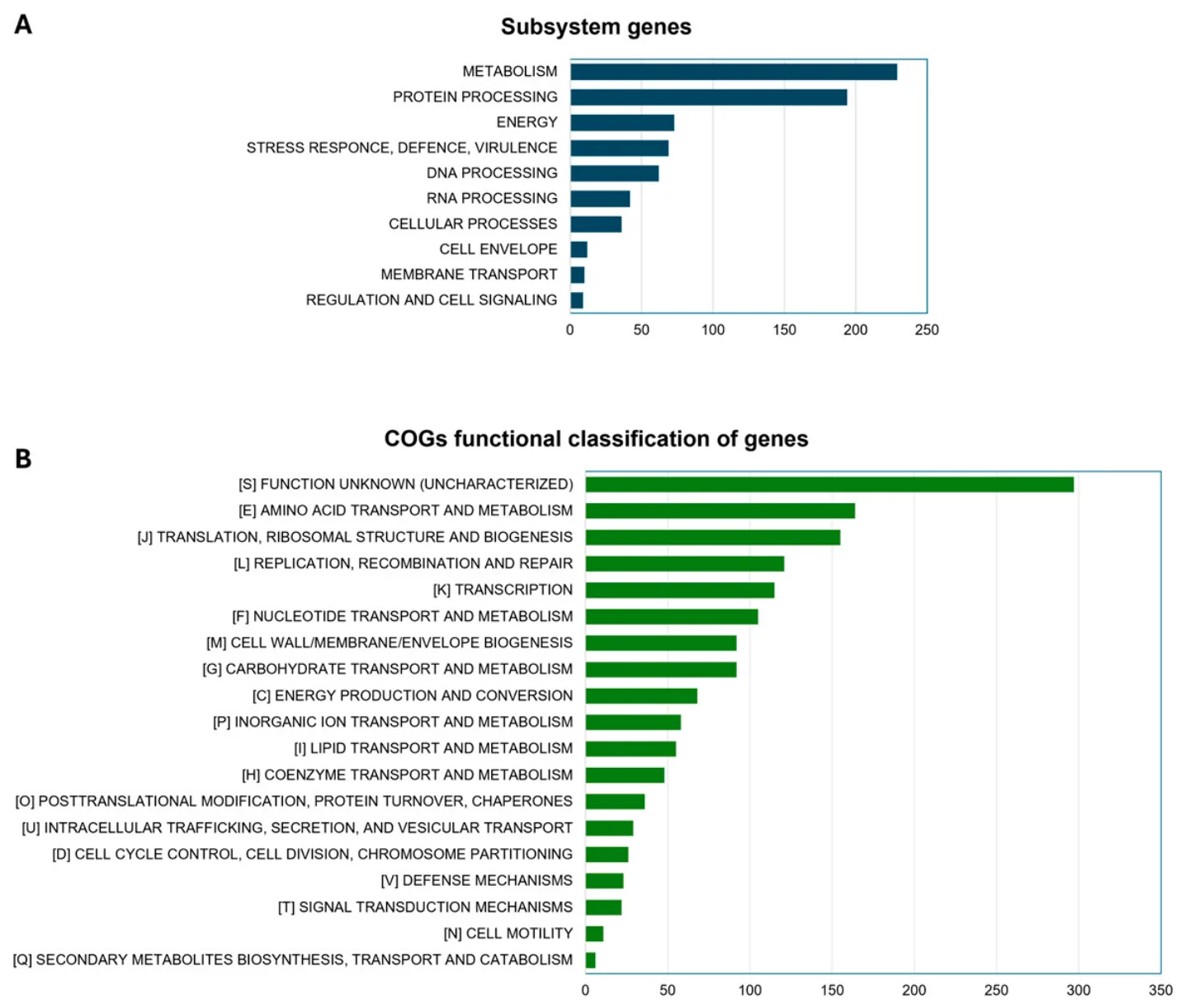
(**A**). Subsystems and associated genes counted in the *L. mucosae* CRL2069 genome. (**B**) COGs functional classification of genes annotated in *L. mucosae* CRL2069.

Analysis of the annotated genome showed a total of 1264 protein-coding genes with assigned functions and the identification of 613 hypothetical proteins. Most genes of *L. mucosae* CRL2069 belonged to the metabolism subsystem (n = 299), followed by protein processing (n = 194), energy (n = 73), stress response/defence/virulence (n = 70), DNA processing (n = 62), and RNA processing (n = 42) as summarized in Figure 1A.

According to eggNOG-mapper functional classification the largest proportion of CRL2069 genes corresponded to translation and ribosomal structure/biogenesis (11%), followed by amino acid transport and metabolism and replication/recombination/repair (9%), transcription (8%), nucleotide metabolism/transport (7%), while wall/membrane/envelope biogenesis and carbohydrate transport and metabolism accounted for 6% each (Figure 1B). The overall distribution of functional categories was comparable to that reported for strain *L. mucosae* LM1, in which the most abundant categories included replication/recombination/repair (8.5%), amino acid transport and metabolism (7.7%), translation and ribosomal biogenesis (6.4%), transcription (5.6%), carbohydrate metabolism/transport (5.6%), and cell wall/membrane biogenesis (5.0%) [61]. Overall, the functional profiles suggest that *L. mucosae* CRL2069 is metabolically versatile, with a broad repertoire of genes supporting its ability to survive in hostile environments such as the GIT.

#### 3.1.2. Defence Systems and Genomic Stability

A total of 12 genomic islands were identified in *L. mucosae* CRL2069 (Supplementary Table S2). These GIs include genes involved in carbohydrate metabolism as glycosyltransferases and polysaccharide biosynthesis proteins (islands 1 and 3), clustered regularly interspaced short palindromic repeat (CRISPR)-associated proteins (island 8), and a bacteriophage exclusion (BREX) system and a type II toxin–antitoxin module (HicA–HicB) in island 9. Two CRISPR (clustered regularly interspaced short palindromic repeat) loci were identified in the *L. mucosae* CRL2069 genome, one containing a single spacer and the other comprising 22 spacers (Table 2). CRISPRCasFinder also detected three Cas gene clusters. The largest encodes a complete Type I-C system, characterized by the presence of *cas1*, *cas2*, *cas3*, *cas4*, *cas5c*, *cas7c*, and *cas8c*, the latter being a diagnostic marker of subtype I-C [62]. Two additional clusters contained only *cas3* genes and may represent remnant or incomplete CRISPR-Cas loci.

Similarly, two CRISPR-Cas loci (Type I and II) were reported in *L. mucosae* LM1 [61], while a survey across 44 *L. mucosae* strains identified four distinct CRISPR-Cas loci (type I-E, I-C, II-A and III-A) (Jia et al., 2020 [58]). In contrast, only CRISPR arrays, without associated *cas* genes, were detected in *L. mucosae* CRL573 [55], suggesting that the presence and functionality of CRISPR-Cas systems can vary considerably even among closely related strains. This genetic diversity underscores the dynamic nature of these defence systems and their role in strain-specific adaptation to environmental pressures such as phage predation. LAB harbour a higher number of CRISPR-Cas systems compared to other bacteria, being present in approximately 63% of lactobacilli [63]. These loci contribute to genetic stability and adaptive immunity by preventing infection and bacteriophage conjugation and natural transformation with foreign nucleic acid fragments [64,65].

In addition to CRISPR-Cas, BREX and toxin–antitoxin (TA) modules were identified as defence systems in the CRL2069 genome. The BREX system has been recently described as a phage resistance mechanism in which bacterial DNA is methylated by a methyltransferase (BrxX/PglX), thereby preventing replication and integration of unmethylated phage DNA [66,67]. Similar systems have been reported in *Bacillus*, *Escherichia coli*, and LAB, including *Lacticaseibacillus casei* Zhang, which carries an eight-gene BREX cassette [68]. TA systems, consisting of a stable toxin and an unstable protein or RNA antitoxin, are widespread among bacteria and archaea. Under normal conditions, the antitoxin neutralizes the toxin; however, under stress, the antitoxin is degraded, releasing the toxin, which modulates various cellular processes [69,70]. The presence of these self-defence and immunity systems in *L. mucosae* CRL2069 likely enhances its resistance to stressors and foreign genetic elements, contributing to its genomic stability and safety profile. These attributes are consistent with a strain capable of surviving and competing effectively in the intestinal environment, supporting its potential as a probiotic.

#### 3.1.3. Mobile Genetic Elements and Niche Specialization

The CRL2069 genome contains various integrases/recombinases and 28 transposases (Supplementary Table S3). DNA transposases catalyze the movement of discrete DNA from one location in the genome to another, contributing to genetic plasticity and promoting genetic diversity within the species and adaptability to changing environmental conditions [71]. Interestingly, no intact prophage regions were predicted by PHASTEST, and identified remnants were classified as non-functional due to an insufficient number of genes.

The combination of a complete Type I-C CRISPR-Cas system, the absence of intact prophages, differently compared to more broadly distributed and multihabitat species, such as *Limosilactobacillus fermentum* CRL2085 and other [72,73], and the limited number of functional mobile genetic elements observed in *L. mucosae* CRL2069 may reflect adaptation to a relatively stable host-associated environment. Such genomic features could contribute to the maintenance of genetic integrity by limiting the acquisition of foreign DNA, while still allowing evolutionary diversification through selected mobile genetic elements.

#### 3.1.4. Resistome Analysis and Safety Assessment

Analysis of the resistome of strain CRL2069 using the CARD database identified the presence of the *tet*(W) gene. However, bioinformatic analyses indicate that the *tet*(W) gene (UIK88_02115) lacks the potential for horizontal gene transfer since it is chromosomally integrated and flanked upstream by an essential metabolic maintenance gene encoding a HesB/YadR/YfhF family protein (UIK88_02110) and downstream by a serine hydrolase (UIK88_02125).

### 3.2. Phylogenomic and Comparative Genomic Analysis

Phylogenomic analysis confirmed the taxonomic placement of strain CRL2069 within the *L. mucosae* species (Supplementary Figure S1). Comparative analysis was performed on a total of 54 *L. mucosae* genomes (Supplementary Table S1) using Pan-Explorer, which identified a pangenome size of 6908 gene clusters, comprising 990 core, 3704 dispensable, and 2215 strain-specific genes.

**Figure 2:**
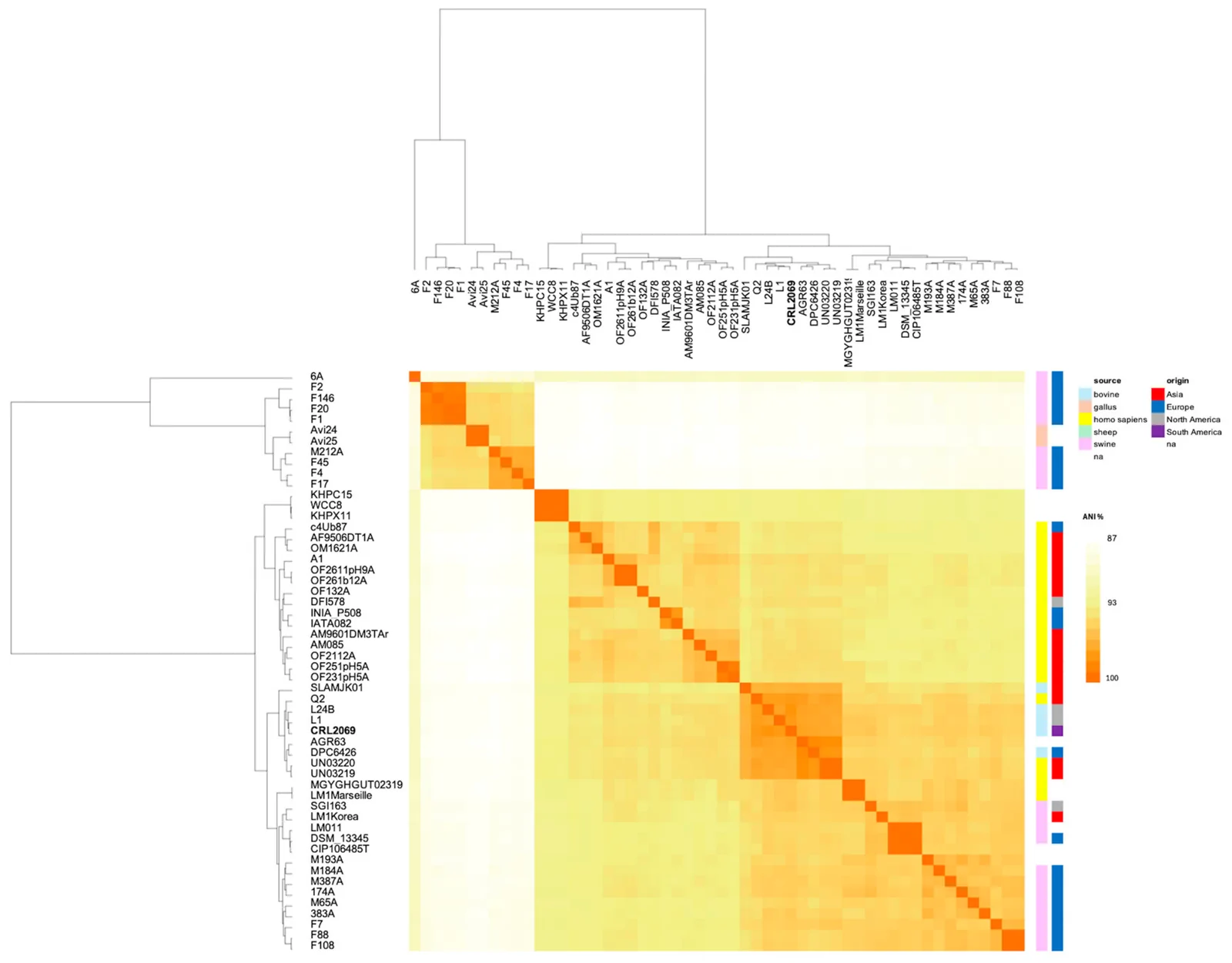
Whole-genome sequence comparisons and heatmap of the Average Nucleotide Identity among *L. mucosae* strains. Isolation source and geographical origin are indicated by a square box close to the strain name, according to the legend.

The genetic divergence, based on the ANI (Average Nucleotide Identity), is shown in Figure 2. According to values reported in Supplementary Table S4, the mean pairwise ANI percentage across all genomes is 94.09%; specifically, the *L. mucosae* L24B genome was found to be the closest relative to *L. mucosae* CRL2069, with an ANI value of 99.09%.

The analyzed genomes formed two clearly separated lineages. The first lineage comprised 11 strains sharing an average ANI of 96.23%, whereas the second, larger lineage comprised 43 strains with a mean ANI of 95.21%. Notably, ANI values between strains belonging to the two lineages ranged from approximately 87% to 92%, well below the generally accepted species delineation threshold of 95–96%, indicating a remarkable genomic divergence and suggesting that some publicly available genomes currently assigned to *L. mucosae* may represent taxonomically distinct lineages.

Within this second lineage, CRL2069 clustered with six closely related strains (L24-B, AGR63, L1, Q2, SLAMJK01, and DPC6426), all isolated from bovine rumen or fecal samples collected in different geographical regions (North America, Europe, and Asia).

The phylogenomic and pan-genomic analyses supported the ANI-based relationships and suggested that host source, rather than geographical origin, contributed to the observed population structure. Although strains from different hosts were interspersed throughout the phylogeny, several small subclades were enriched in isolates recovered from the same host, particularly bovine or swine. Consistent with this pattern, CRL2069 clustered with bovine-associated strains despite their diverse geographical origins, supporting the hypothesis that adaptation to the bovine gastrointestinal environment has contributed to the evolution of this lineage. Likewise, the accessory genome tree (Supplementary Figure S2) revealed a similar organization, whereas the highly divergent lineage identified by ANI, comprising strains isolated from swine (F45, F17, F4, M212A, F2, F20, F1, and F146) and poultry (Avi24 and Avi25), remained clearly separated from the rest of the *L. mucosae* population. This distribution is consistent with the hypothesis that ecological adaptation, rather than geographic separation, has played an important role in shaping the genomic diversity of *L. mucosae*. The occurrence of strains isolated from similar hosts in distantly related clades further suggests that host-associated adaptive traits have been maintained in distinct evolutionary lineages.

### 3.3. In Silico Carbohydrate-Active Enzymes (CAZymes) and Pattern of Carbon Source Utilization

#### 3.3.1. Genomic Prediction of CAZymes

Genome annotation identified 45 genes encoding putative CAZymes, mainly belonging to the glycosyltransferases (GTs) family (25 genes), followed by glycoside hydrolases (GHs, 20 genes), two carbohydrate-binding modules (CBMs), and one auxiliary activity (AA) enzyme (Table 3), suggesting robust metabolic specialization toward carbohydrate utilization.

A defining characteristic of beneficial gut bacteria is their capacity to degrade a broad range of carbohydrates, a feature largely mediated by diverse glycoside hydrolase (GH) families. The presence of genes related to carbohydrate metabolism enhances the strain’s survival, competitiveness, and persistence within gastrointestinal microhabitats, while promoting interactions with the host [39,74].

The 25 GT-encoding genes identified in the genome were distributed across 11 families (GT2, GT4, GT8, GT28, GT32, GT51, GT111, GT113, GT119, GT125, GT129), with the most abundant being GT2 (7 genes), GT4 (6 genes) and GT8 (2 genes), whereas the identified GHs were diverse and distributed across 14 families (GH2, GH3, GH8, GH13, GH25, GH31, GH32, GH36, GH42, GH43, GH51. GH65, GH73, GH120).

Among GHs, GH73 was the most represented family (4 genes), followed by GH13, GH42, and GH43 (2 genes each). Glycoside hydrolases (EC 3.2.1) are key enzymes responsible for the degradation of complex carbohydrates, catalyzing the hydrolysis of glycosidic bonds between carbohydrate moieties or between a carbohydrate and a non-carbohydrate group. Four GH73 family genes were present in CRL2069. Two of them were predicted to encode hydrolases involved in the hydrolysis of oligo- and polysaccharides, whereas the remaining two encoded a glucosaminidase and a LysM domain-containing peptidoglycan hydrolase associated with bacterial cell wall remodelling and cell division. The occurrence of these enzymes agrees with findings reported for 93 *L. mucosae* strains mostly isolated from human feces [58] and with *Limosilactobacillus (L.) fermentum* strains from cattle feed rations, food and the human gut [72,75] (Supplementary Figure S3).

Clustering based on the CAZyme repertoire placed CRL2069 close to strain L24-B (bovine feces, USA), Q2 (bovine rumen, China), and AGR63, whose isolation source is unavailable (Supplementary Figure S4). This grouping may reflect the conservation of CAZyme repertoires adapted to the utilization of plant-derived carbohydrates and oligosaccharides generated during forage digestion in the bovine gastrointestinal tract, consistent with the host-associated clustering observed for these strains. In particular, the presence of GH13-family enzymes (alpha-amylases and sucrose phosphorylase), GH31 α-glucosidase, GH65 maltose phosphorylase, GH43 β-xylosidases, GH51 α-L-arabinofuranosidase, and GH36 α-galactosidase, suggests the ability to utilize starch-derived oligosaccharides, raffinose-family sugars, and hemicellulose-derived carbohydrates such as xylans and arabinoxylans, which are common components of cattle diets. These observations are consistent with the host-associated clustering observed for these strains.

Genes belonging to the GH13 superfamily were also identified, similar to what was reported for *L. mucosae* strains from wild boars and other publicly available genomes [59]. These enzymes are typically surface-associated and often contain repeat sequences that facilitate binding to the cell wall (https://www.cazypedia.org/index.php/Glycoside_Hydrolase_Family_13; accessed on 3 December 2025). In particular, two proteins classified within the sub-families GH13_18 and GH13_39 were predicted in the *L. mucosae* CRL2069 genome. The former enzyme, annotated as sucrose phosphorylase (UIK88_01400), catalyzes the phosphorolysis of sucrose to yield α-glucose 1-phosphate and fructose. Sucrose phosphorylases may also participate as transglucosylases for the synthesis of valuable glycosides and sugars through the transfer of the glucosyl group from sucrose [76]. The second, annotated as the α-amylase family glycosyl hydrolase (UIK88_08805), catalyzes the hydrolysis of alpha-1,4-glucosidic bonds in starch and related alpha-glucans.

In addition to GH73 and GH13, the CRL2069 genome encodes β-galactosidase (GH42 family) and enzymes involved in the degradation of long-chain carbohydrates (GH43 family). Genes belonging to these families were also identified in the other *L. mucosae* strains as reported by Jia et al. [58] and Kavanova et al. [59].

Two carbohydrate-binding modules (CBMs) were also detected in the CRL2069 genome. These modules enhance enzyme–substrate affinity by binding specifically to carbohydrate structures, thereby increasing the efficiency of carbohydrate degradation.

The 25 GTs identified in the genome of *L. mucosae* CRL2069 participate in the biosynthesis of disaccharides, oligosaccharides, and polysaccharides, as well as in protein glycosylation and the formation of numerous biologically important natural products. They catalyze the transfer of activated sugar donors to specific acceptors, which may include mono-, di-, or oligosaccharides, proteins, lipids, nucleic acids, or small molecules [77,78]. In CRL2069, the most abundant GT families were GT2 (seven genes) and GT4 (six genes), which are mainly involved in the synthesis of EPS, consistent with the findings of Jia et al. [58]. It was reported that the alteration or deletion of specific glycosyltransferase genes in *Lactobacillus johnsonii* FI9785 drastically reduced or abolished EPS production, confirming the essential role of these enzymes in their biosynthesis; such mutations also resulted in growth defects, which were restored through genetic complementation [79].

#### 3.3.2. Phenotypic Profiling of Carbon Source Utilization

**Figure 3:**
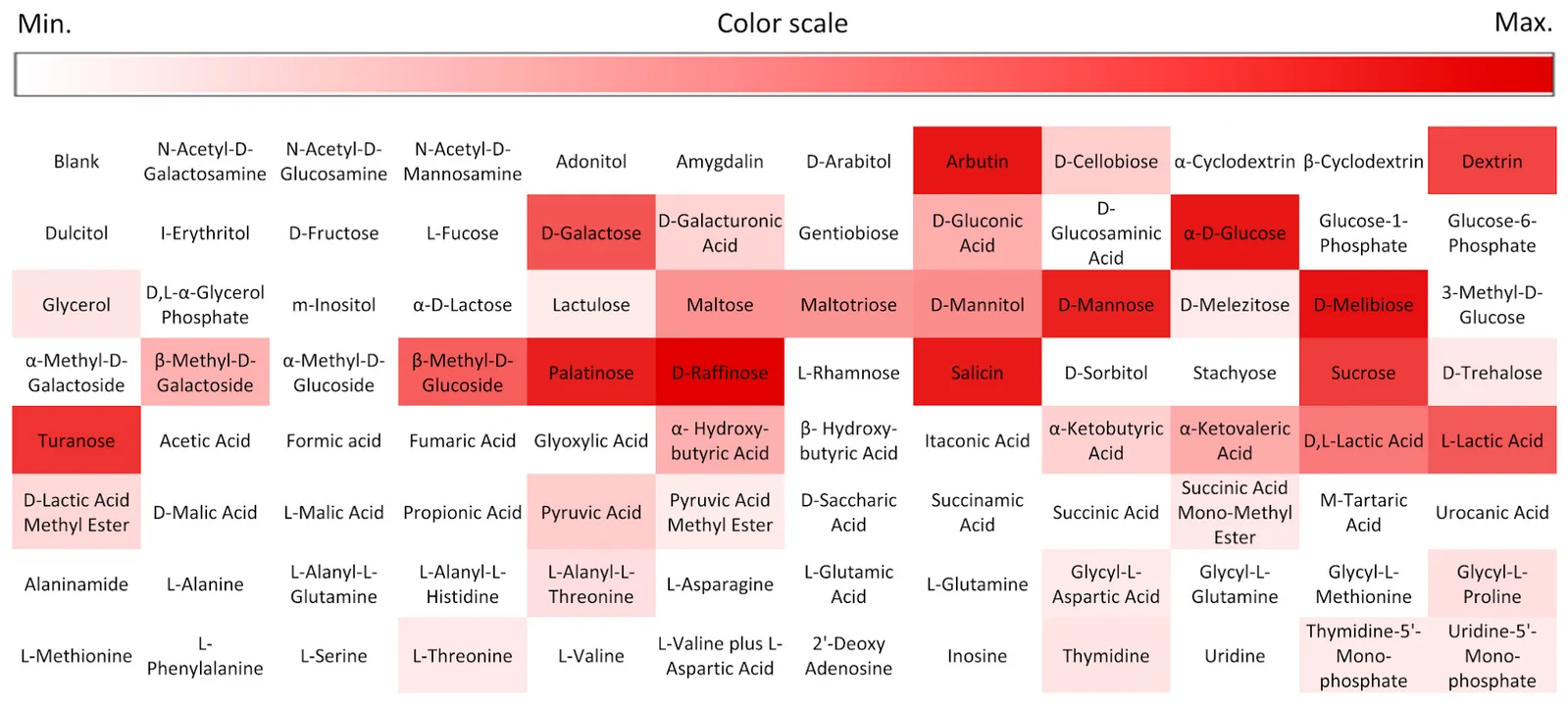
Carbon source utilization or oxidation by *L. mucosae* CRL2069 using an AN MicroPlate (Biolog Inc., Hayward, CA, USA). The colour scale indicates the metabolism of each carbon source ranging from white (no utilization or oxidation) to dark red (maximum utilization or oxidation).

To experimentally validate the carbohydrate utilization potential predicted by genome analysis, carbon source utilization was evaluated using the Biolog AN microplates system (Figure 3). CRL2069 used 39 out of 95 tested substrates. The highest levels of utilization were observed for arbutin, α-D-glucose, D-mannose, D-melibiose, D-raffinose, and salicin, while moderate consumption was detected for dextrin, D-galactose, palatinose, sucrose, and turanose. When the fermentation ability of CRL2069 was compared to that of 93 *L. mucosae* strains evaluated by Jia et al. [58], distinct utilization profiles were noted. While most strains in that study fermented D-lactose, fucose, and D-xylose, CRL2069 was unable to metabolize these substrates. Conversely, CRL2069 successfully utilized cellobiose, D-mannitol, and D-mannose, which were not consumed by the strains reported by Jia et al. [58]. These discrepancies support the hypothesis that carbohydrate utilization patterns in *L. mucosae* can be strain-specific and partially independent of the habitat or host [58]. The phenotypic profile was broadly consistent with the CAZyme repertoire identified in the genome, supporting the predicted capacity of CRL2069 to metabolize a diverse range of plant- and host-derived carbohydrates.

#### 3.3.3. Metabolism of Phytochemicals and Plant-Derived Carbohydrates

Lactobacilli possess a strain- or species-specific ability to metabolize disaccharides such as cellobiose [Glu-β-(1 → 4)-d-Glu], trehalose [Glu-α-(1 → 1)-d-α-Glu], and the α-d-glucosyl-d-fructose isomers turanose and palatinose [80], which, for *L. mucosae* CRL2069, was confirmed by the AN Biolog assay. Interestingly, despite the genomic annotation of GH42 β-galactosidase, CRL2069 was unable to utilize lactose (AN Biolog), indicating that the presence of a GH42 β-galactosidase gene alone is insufficient to predict lactose utilization, which may depend on gene regulation, transporter availability or growth conditions; this is consistent with the reported low or absent activity of β-galactosidase in several *Lactobacillus* and *Carnobacterium* species under certain conditions [80]. Furthermore, although D-mannose, salicin, D-mannitol and cellobiose were utilized by CRL2069 to varying degrees, these sugars could not be consumed by *L. mucosae* strains reported by Jia et al. [58]. The Biolog AN assay showed that arbutin, D-glucose, D-mannose, D-melibiose, D-raffinose and salicin were degraded at the highest level by *L. mucosae* CRL2069. Arbutin (β-glucoside derived from hydroquinone) is a natural phytochemical that can be hydrolyzed by β-glycosidases and has been reported to improve gut development and serum lipids in the host. The potential of *Lactobacillus intestinalis* to proliferate by utilizing arbutin to increase biological activity has also been reported [81].

High utilization of D-mannose was observed, which is a component of hemicellulose and glycoproteins in the plant cell wall and is abundantly found in forages and cereal grains. Among dietary carbohydrates, raffinose (α-Gal-(1/6)-α-Glu-(1/2)-β-Fru), a trisaccharide widely distributed in plants (soybeans, oil crops, and fruits), has been considered an anti-nutritional factor. Several lactobacilli have been reported to degrade raffinose into digestible carbohydrates, which provides a promising solution for both raffinose removal and better utilization of raffinose in GIT. Two pathways (via levansucrase or via α-galactosidase) have been described for raffinose utilization [82]. Extracellular levansucrases cleave the bond between fructose and glucose in raffinose to yield fructose and melibiose, which are then transported into cells and hydrolysed by intracellular α-galactosidase (MelA). Alternatively, the catabolism of raffinose by α-galactosidase may require direct intracellular transport followed by hydrolysis into sucrose and galactose; subsequently, sucrose phosphorylase can convert into α-d-glucose-1-phosphate and β-d-fructose. This metabolic pathway has been reported for several probiotic lactobacilli [82].

Salicin, a β-alcoholic phytochemical, was also efficiently consumed by CRL2069. In the intestine the gut microbiota hydrolyses the β-glycosidic bond of salicin, releasing saligenin and glucose. Following oxidation in the blood or liver, saligenin is converted into pharmacologically active salicylic acid (salicylate) via the chorismate biosynthetic pathway [83].

In addition, CRL2069 exhibited a moderate utilization of dextrin. Dextrins represent intermediate products of ruminal starch digestion, a process driven by amylase activity. These low-molecular-weight oligosaccharides are used as a prebiotic (non-digestible compound) facilitating the growth and activity of probiotics, inhibiting pathogens and supporting the immune system [84].

The ability of CRL2069 to metabolize such a diverse array of carbohydrates highlights its metabolic versatility and supports its adaptation to the bovine gastrointestinal environment. Efficient utilization of these substrates enhances its probiotic potential, particularly considering that many of these sugars are prevalent in cattle feed ingredients, such as forages and plant-derived byproducts.

### 3.4. Genomic Determinants Associated with Probiotic and Nutritional Functions

Putative genes associated with functions relevant to probiotic activity were identified in *L. mucosae* CRL2069 (Supplementary Table S5). These included genes involved in stress tolerance, protein quality control, adhesion, biofilm formation, exopolysaccharide biosynthesis, and vitamin metabolism, representing functional categories that may contribute to bacterial persistence and adaptation within the gastrointestinal environment.

#### 3.4.1. Stress Response and Protein Homeostasis

It is known that the survival and persistence of administered probiotics is highly affected by the harsh conditions of the GIT (acid exposure, oxidative stress, bile salts, osmotic fluctuations, and nutrient limitation). Thus, different stress response mechanisms have been developed, involving modification of the cell envelope and membrane lipids, alteration of metabolic pathways, and over-expression of stress proteins for macromolecule repair [85].

*Core Chaperonin Systems (Hsp60 and Hsp70 Families)*

Several genes associated with stress response were predicted in the CRL2069 genome (Supplementary Table S5), including *groES* (UIK88_07435) and *groEL* (UIK88_07440) from the Hsp60 family [86], and *dnaK* (UIK88_05650) and *dnaJ* (UIK88_05645), which encode conserved chaperone systems involved in protein folding and stress tolerance in bacteria [86].

*Oxidative Stress Defence and Redox Homeostasis Mechanisms*

In addition, several genes involved in oxidative stress response and redox homeostasis were identified in the *L. mucosae* CRL2069 genome, including putative thioredoxin *trxA*, thioredoxin-disulfide reductase *trxB* gene and the genes *hslO* (UIK88_07170), encoding a redox-regulated molecular chaperone of the Hsp33 family, and *tpx* (UIK88_08365), encoding a thiol-specific peroxidase. Similar systems have been reported in other probiotic lactobacilli, including *L. fermentum* CRL2085 and *Lactobacillus johnsonii* CNCM I-4884 [72,87]. Together, these genes may contribute to oxidative stress tolerance and maintenance of cellular redox balance.

*The Clp Proteolytic Machinery and Protein Quality Control*

Genes coding for the Clp (caseinolytic protease) family proteins were also identified in CRL2069. Among them, the genes *clp*X (coding for the ATP-dependent protease ATP-binding subunit) and *clp*P (coding for the ATP-dependent Clp endopeptidase proteolytic subunit) encode components of Clp ATPase-ClpP proteolytic complexes, which play indispensable roles in cellular protein quality control systems by refolding or degrading damaged proteins in both stressed and non-stressed cells, particularly in LAB and other low-GC bacteria [88,89].

#### 3.4.2. Acid, Bile Salt, Alcohol and Extreme Temperature Tolerance

Resistance to gastrointestinal stressors is essential for probiotic colonization [87]. In CRL2069, two genes, the Na+/H+ antiporter *nhaC* (UIK88_04705) and a DASS (Divalent Anion/Sodium Symporter) family sodium-coupled anion symporter (UIK88_02205), were annotated, in agreement with data reported for *L. mucosae* DSM 13345, LM1, and F146 strains [56,90,91]. These transport systems may contribute to intracellular pH homeostasis and adaptation to gastrointestinal environments.

*Proton extrusion and Acidic Stress adaptation*

Eight genes encoding the F0F1-ATPase complex (including *atp*D, *atp*A, *atp*H, *atp*F, *atp*E, *atp*B (UIK88_08220 to UIK88_08185)) were also identified. The F0F1-ATPase complex is considered essential for acid tolerance in lactic acid bacteria [92]. Additionally, two genes encoding glucose-6-phosphate isomerase, which convert glucose-6-phosphate to fructose-6-phosphate, were identified in the CRL2069 genome. These enzymes are involved in acid and bile tolerance, acting as acid-shock proteins, and can also function as moonlighting adhesion proteins, as observed in *L. acidophilus* and *L. crispatus* [93,94]. The presence of multiple acid resistance-related genes may contribute to CRL2069 adaptation to acidic GIT environments, consistent with other *L. mucosae* strains [55,56,90,91].

*Bile Tolerance and Stress Response Mechanisms*

A total of 18 genes related to bile tolerance were identified, including a malolactic enzyme (UIK88_04550) and arginine deiminase (ADI) pathway genes, such as arginine deiminase (UIK88_06985) and an arginine deiminase family protein (UIK88_04280). Stress response regulators, such as a bifunctional (p)ppGpp synthetase/hydrolase (UIK88_05600) and a GTP pyrophosphokinase family protein (UIK88_02900), were also detected. The malolactic enzyme (MleS) converts malate into lactate and CO_2_, consuming protons and contributing to acid resistance. Although the disruption of the malolactic pathway in *Streptococcus mutans* leads to a loss of acid tolerance [95], MleS enhances survival under bile- and acid-associated stress [96]. Similarly, the ADI pathway contributes to acid stress resistance by converting arginine into ornithine, ammonia, and CO_2_, generating ATP and increasing intracellular pH through ammonia production [97].

The presence of genes governing (p)ppGpp suggests the ability of CRL2069 to activate conserved stringent response mechanisms during nutrient limitation and other environmental stresses by controlling DNA replication, protein synthesis, and energy metabolism [98].

*Energetic Efficiency via the Phosphotransferase System (PTS)*

Moreover, four putative genes encoding components of the phosphotransferase system (PTS) were predicted in the CRL2069 genome. Several sugar-specific PTS components were identified, including the mannose/fructose/sorbose family transporter subunit IID (UIK88_04455) and subunit IIC (UIK88_04450) genes, as well as two PTS sugar transporter subunit IIC (UIK88_06015 and UIK88_07800) genes.

The PTS represents a major active sugar transport system in bacteria, offering the advantage of being energetically more efficient than other active transport systems, since it is coupled with substrate-level phosphorylation [99]. The PTS also contributes to bile tolerance by regulating carbon uptake, biofilm formation, and stress response [100]. Similar PTSs are present in *L. fermentum* CRL2085, ATCC 23271, and LAB- [72,101,102].

*Pyruvate Metabolism and Thermal Stress Response*

In LAB, conversion of pyruvate to lactate is essential for maintaining NAD^+^ regeneration and sustaining glycolytic flux under fermentative conditions [103]. Five genes encoding L-LDH were identified in the genome of CRL2069 (UIK88_01860, UIK88_02200, UIK88_04970, UIK88_05630, UIK88_07535). The presence of multiple *ldh* genes suggests substantial lactic acid production, which may contribute to pathogen inhibition and the establishment of a favourable environment for beneficial bacteria. Finally, the *L. mucosae* CRL2069 genome contains two genes associated with resistance to extreme temperatures: one encoding a Cold-Shock Protein (CspA) (UIK88_09115) and another encoding a protein of the small heat-shock protein (Hsp20) family (UIK88_06270).

#### 3.4.3. Adhesion and Host Interaction

Adhesion to the intestinal mucosa is a key trait of probiotics, supporting colonization and interaction with host cells [87]. In *L. mucosae*, this trait is particularly relevant due to the ecological specialization of the species toward mucosal environments. In this regard, a gene encoding a putative LPXTG-anchored surface protein (UIK88_05770) was identified in the CRL2069 genome. LPXTG-anchored proteins are commonly associated with bacterial adhesion to host tissues and persistence within mucosal environments. Such proteins have also been described in multiple *L. mucosae* strains, including CRL573 [55], LM1 [20], and DSM13345 [90].

*Cell wall Remodelling and Mucin-Binding Proteins*

Three genes encoding NlpC/P60 family proteins (UIK88_00330, UIK88_00340, UIK88_01845) were also identified. These peptidoglycan hydrolases, present in many bacteria [104], contribute to cell wall remodelling, cell division, and biofilm formation. NlpC/P60 proteins have also been implicated in adhesion and persistence of lactic acid bacteria. Similar genes were also reported in *L. mucosae* F108 and F146 strains [91].

Furthermore, two genes encoding MucBP domain-containing surface proteins (UIK88_00505 and UIK88_00555) were identified in CRL2069. These surface proteins share homology with the streptococcal GspB-like adhesin and are characterized by glycosylation and a selective affinity for sialic acid, which allows *L. mucosae* to adhere effectively to intestinal mucins [61]. The presence of these genes supports a fundamental niche specialization of CRL2069 toward the bovine intestinal environment.

*Immunobiotic Potential in the Bovine Host*

CRL2069 has previously demonstrated immunomodulatory effects when evaluated in bovine intestinal epithelial cells (BIEs). In this model, the strain modulated innate immune responses triggered by both TLR3 and TLR4 through the regulation of three negative regulators, leading to a reduced expression of pro-inflammatory cytokines and chemokines [11]. This regulatory activity positions *L. mucosae* CRL2069 as a promising immunobiotic candidate for protecting the bovine host against intestinal inflammatory damage.

#### 3.4.4. Biofilm Formation and Surface Adaptation

Thirteen genes potentially associated with biofilm development and surface adherence were predicted in the *L. mucosae* CRL2069 genome. These included genes putatively coding for the quorum sensing-related enzyme S-ribosyl homocysteine lyase LuxS (UIK88_02300), LCP family proteins (UIK88_06175, UIK88_07770, UIK88_00850), and several components of the type IV competence system. The latter included the ComE operon protein 2 (*comEA*, UIK88_09250), one helicase C-terminal domain-containing protein (*comFA*, UIK88_08865), the type II secretion system component ComGB (*comGB*, UIK88_08605, UIK88_08595), the DNA internalization-related competence protein ComEC/Rec2 (*comEC*, UIK88_09255), and the competence type IV pilus major pilin ComGC (*comGC*, UIK88_08600). The *comGC* gene encodes the major pilin subunit of type IV pili, which contribute to several key functions, including DNA uptake, biofilm formation, cell adhesion, and motility [72,105]. In addition, LytR–CpsA–Psr (LCP) family proteins play a critical role in cell envelope synthesis and assembly [106]. Bacterial communication through the quorum sensing (QS) system, which relies on diffusible signalling molecules, is essential for biofilm development. The enzyme LuxS plays a central role in the production of extracellular polysaccharides and cell adhesion by catalyzing the synthesis of the autoinducer-2 (AI-2) signalling molecule, whose concentration increases with cell density. Beyond its role in quorum sensing, LuxS also contributes to the regulation of bacterial growth [107].

*Biofilm Formation and Colonization Potential*

The genomic features identified in *L. mucosae* CRL2069 suggest a potential ability to develop biofilm-associated lifestyles, which may contribute to persistence and adaptation within the bovine gastrointestinal environment. In CRL2069, the presence of genes involved in competence-associated surface structures, cell envelope organization, and quorum sensing, including *comGC*, *comEA*, *comFA*, LCP family proteins, and *luxS*, provides a genomic basis for potential biofilm development and host adaptation. These features may contribute to the persistence of CRL2069 in the bovine intestinal tract, supporting its previously demonstrated immunomodulatory activity in bovine intestinal epithelial cells [11].

#### 3.4.5. EPS Biosynthesis

Exopolysaccharides (EPSs) contribute to bacterial adaptation by protecting cellular integrity under adverse environmental conditions and increasing resistance against antibiotics and phages [72]. They may also provide health benefits to the consumer, including immunostimulatory, antitumor, and cholesterol-lowering effects [108]. Genomic analysis of CRL2069 identified several genes potentially involved in the synthesis of nucleotide-sugar precursors associated with EPSs and cell wall biosynthesis, including *galE* (UIK88_07310), galU (UIK88_07525), *pgmB* (UIK88_08950), *glmU* (UIK88_07015), *glmM* (UIK88_07740), and *gndA* (UIK88_01570). These genes participate in the biosynthesis of UDP-glucose, UDP-galactose, and UDP-N-acetylglucosamine, which are important precursors for polysaccharide production and other cellular processes [108,109,110,111].

Although these genomic features indicate that CRL2069 possesses the enzymatic machinery required for the synthesis of EPS precursors, phenotypic evaluation showed that this strain did not produce detectable EPS under the conditions tested. This discrepancy highlights that the presence of biosynthetic genes alone does not necessarily predict EPS production. In lactic acid bacteria, EPS synthesis is often highly dependent on environmental and culture conditions, including carbon source availability, growth phase, pH, temperature, and regulatory mechanisms. Furthermore, the genes identified in CRL2069 are mainly involved in the synthesis of nucleotide-sugar precursors that can be used for EPS production as well as for other cellular processes, and their presence alone does not demonstrate the existence of a fully functional and actively expressed EPS biosynthetic pathway. Therefore, CRL2069 may retain the genetic potential for EPS synthesis, although this phenotype was not expressed under the experimental conditions evaluated in the present study.

#### 3.4.6. Folate Biosynthesis

Genomic analysis revealed the presence of key genes involved in the folate biosynthetic pathway (*folE*, *folB*, *folK*, *folP*, *folC*, *folA*), an essential cofactor required by all living cells [61]. Folate production has previously been reported in several LAB, including *Lactobacillus delbrueckii* subsp. *bulgaricus*, *L. plantarum*, and *L. acidophilus*, which are commonly incorporated into dairy products to enhance their folate content. In addition, genes associated with folate biosynthesis have been identified in *L. fermentum* KUB-D18 and *L. mucosae* LM1 [39,61]. In *L. mucosae* LM1, folate biosynthesis was associated with the ability to utilize para-aminobenzoic acid (PABA), a precursor derived from the shikimate pathway [61]. Consistent with the genomic prediction, *L. mucosae* CRL2069 was found to produce folate under the conditions tested (Supplementary Figure S5), confirming the functional expression of the annotated biosynthetic pathway. When considering potential applications in animal nutrition, supplementation with folate-producing probiotics may represent an attractive strategy to complement diets, as folate-producing strains may contribute to the vitamin supply either through release into the feed or via biosynthesis within the gastrointestinal tract of the host [112].

### 3.5. In Silico Analysis of Bacteriocin Biosynthetic Gene Clusters

To investigate the antimicrobial potential of *L. mucosae* CRL2069, its genome was screened for bacteriocin biosynthetic gene clusters using BAGEL4 (BActeriocin GEnome mining tooL 4). A single gene encoding a lanthionine synthetase LanC family protein (UIK88_00475) was identified. However, analysis of the genomic region surrounding *lan*C revealed the absence of other core biosynthetic genes required for lantibiotic production, such as the precursor peptide gene (*lanA*) and the dehydratase gene (*lanB* or *lanM*). Moreover, a gene encoding an integrase core domain-containing protein (UIK88_00465) is located immediately upstream, *lan*C, suggesting that this genomic region may have originated through horizontal gene transfer. Therefore, the identified locus is unlikely to constitute a complete and functional lantibiotic biosynthetic cluster and may represent a remnant of an ancestral bacteriocin locus that has undergone partial gene loss during evolution. Confirming the genomic prediction, CRL2069 did not display any bacteriocin production, as reported by Maldonado et al. [15].

## 4. Conclusions

The present study provides a comprehensive genomic and phenotypic characterization of *Limosilactobacillus mucosae* CRL2069, a strain isolated from feedlot cattle feces, highlighting several traits associated with probiotic potential.

Genome analysis revealed the presence of genes involved in acid, bile salt, oxidative, and osmotic stress tolerance, supporting its ability to survive and persist within the bovine GIT. The combined genomic and phenotypic analyses demonstrated a broad capacity to utilize an array of diverse carbohydrates, particularly those present in cattle feed such as grains (maize and soybean) and forages, as well as the ability to produce folate, suggesting a potentiality to enhance animal nutritional, health and productive performance. These genomic attributes, together with the presence of genes associated with adhesion, surface adaptation, and biofilm development, support the capacity of CRL2069 to colonize and persist in the intestinal environment, traits that appear to be shared by several *L. mucosae* strains.

In addition, the lack of horizontally acquired antimicrobial resistance and virulence genes validated the safety of this strain, confirming its safety and suitability. Taken together, the properties of *L. mucosae* CRL2069 identify this strain as an attractive candidate for use as a probiotic in commercial feedlot systems.

## Figures and Tables

**Table 1:** Genomic features of *L. mucosae* CRL 2069.

Total length	1,926,883 bp
Number of scaffolds	26
Largest contig	465,792 bp
N50	301,383
GC content	46.76
Predicted genes	1888
CDS	1798
tRNA	79
ncRNAs	3
rRNAs	3, 3, 2 (5S, 16S, 23S)
Pseudo genes	28
Coding density %	86.91
Quality	excellent
Completeness	99.99%
Contamination	0.06%

**Table 2:** CRISPR-Cas elements identified in the genome of *L. mucosae* CRL2069.

	Start	End	Evidence Level	Element	Cas Type	Spacer/Gene	Repeat Consensus/Cas Genes
JAXKWS010000004	35,081	35,172	1	CRISPR	-	1	GGTGATGGTGGTGTTCGTCTAGATGATGG
	16,459	17,784	-	Cas cluster	CAS (incomplete)	2	*cas3*
JAXKWS010000007	17,014	18,568	4	CRISPR	-	22	ATTTCAATCCACGCACCCTACAAGAGAGTGCGAC
	18,733	27,539	-	Cas cluster	Type I-C	7	*cas1*, *cas2*, *cas3*, *cas4*, *cas5c*, *cas7c*, *cas8c*
JAXKWS010000020	50,600	52,108	0	Cas cluster	CAS (incomplete)	2	*cas3*

**Table 3:** CAZymes genes identified in *L. mucosae* CRL2069 genome.

Class	Product	Protein ID
AA1	multicopper oxidase domain-containing protein	UIK88_02340
CBM34 + GH13_20	Alpha-amylase (Alpha-amylase starch-binding domain + glycoside hydrolase family 13 protein)	UIK88_00395
		UIK88_06865
CBM91 + GH43_11	beta-xylosidase (Xylan-binding domain + glycoside hydrolase family 43 protein)	UIK88_05150
CE2	carbohydrate esterase family 2	UIK88_01505
CE4	polysaccharide deacetylase family protein	UIK88_05155
CE20	sialate O-acetylesterase	UIK88_05145
GH2	beta-galactosidase	UIK88_04140
GH3	cellulose-degrading glycosyl hydrolase 3	UIK88_07930
GH8	glycosyl hydrolase family 8	UIK88_04350
GH13_18	sucrose phosphorylase	UIK88_01400
GH13_20	glycoside hydrolase family 13 protein	UIK88_00395
		UIK88_06865
GH13_39	alpha-amylase family glycosyl hydrolase	UIK88_08805
GH25	GH25 family lysozyme	UIK88_03970
GH31_1	alpha-glucosidase	UIK88_01880
GH32	sucrose-6-phosphate hydrolase	UIK88_02165
GH36	alpha-galactosidase	UIK88_01535
GH42	beta-galactosidase	UIK88_07705
		UIK88_07940
GH43_11	glycoside hydrolase family 43 protein	UIK88_05150
GH43_24	family 43 glycosylhydrolase	UIK88_05670
GH51_1	alpha-L-arabinofuranosidase C-terminal domain-containing protein	UIK88_05135
GH65	maltose phosphorylase	UIK88_08955
GH73	glycosyl hydrolase family 73	UIK88_00335
		UIK88_01390
		UIK88_02710
		UIK88_06410
GH120	right-handed beta helix domain-containing protein	UIK88_07905
GT2	glycosyltransferase family A protein	UIK88_00305
		UIK88_00355
		UIK88_00805
		UIK88_00810
		UIK88_00825
		UIK88_04355
		UIK88_04850
GT4	glycosyltransferase family 4 protein	UIK88_00445
		UIK88_00450
		UIK88_00815
		UIK88_01050
		UIK88_01055
		UIK88_06435
GT8	glycosyltransferase family 8 protein	UIK88_03400
GT28	undecaprenyldiphospho-muramoylpentapeptide beta-N-acetylglucosaminyltransferase	UIK88_09070
GT32	glycosyltransferase	UIK88_00300
GT51	glycosyltransferase 51/Transpeptidase	UIK88_03000
		UIK88_08835
		UIK88_03405
		UIK88_08770
GT111	β-1,3-galactofuranosyltransferase	UIK88_00280
GT113	galactofuranosyltransferase	UIK88_00295
GT119	peptidoglycan glycosyltransferase FtsW/RodA	UIK88_08250
		UIK88_09220

## Data Availability

The genomic sequencing data datasets generated and analyzed during the current study are available in the DDBJ/ENA/GenBank repository under the accession JAXKWS000000000.

## Supplementary Materials

The following supporting information can be downloaded at https://www.mdpi.com/article/10.3390/microorganisms14102270/s1, Figure S1: Genome-based phylogeny of *L. mucosae* CRL2069. The tree was inferred by using the maximum likelihood method RAxML with progressive refinements. *Lactobacillus oligofermentans* DSM 15707 was used as an outgroup. The tree is drawn to scale. Support values are represented by scaled circles at each node; Figure S2: Accessory-based tree of *L. mucosae* strains. The distance tree was generated by hierarchical clustering from the presence/absence binary matrix of accessory genes. The dendrogram on the left represents hierarchical clustering based on Euclidean distance. Isolation source and geographical origin are indicated by a square box close to the strain name, according to the legend; Figure S3: CAZyme distribution across *Limosilactobacillus mucosae* genomes. Stacked bars represent the number of genes assigned to each CAZy class ((GH), glycosyltransferases (GTs), carbohydrate-binding modules (CBMs), carbohydrate esterases (CEs), and auxiliary activities (AAs)) in individual genomes; Figure S4: Heatmap and hierarchical clustering of *L. mucosae* strains according to their predicted CAZyme repertoire; Figure S5: Growth of *L. mucosae* CRL2069 in FACM medium after 7 consecutive passages. Visible turbidity confirms the strain’s ability to synthesize vitamin B9 de novo; Table S1. Genome list of strains used in this study; Table S2. Genomic Islands predicted in *L. mucosae* CRL2069 genome; Table S3. Transposase predicted in *L. mucosae* CRL2069 genome; Table S4. ANI matrix of *L. mucosae* strains; Table S5. Genes associated with probiotic function in *L. mucosae* CRL2069.
