# Supplementary material for: Genomic Characterization of the Probiotic Limosilactobacillus mucosae CRL2069 Isolated from Feedlot Cattle Feces
Source: Microorganisms. 2026 Oct 6;14(10):2270. doi: 10.3390/microorganisms14102270 (PMC13707948; doi:10.3390/microorganisms14102270)
Supplement: Supplementary file 1 [file microorganisms-14-02270-s001.zip › Supplementary_Fig_1_phylogeny.pdf]

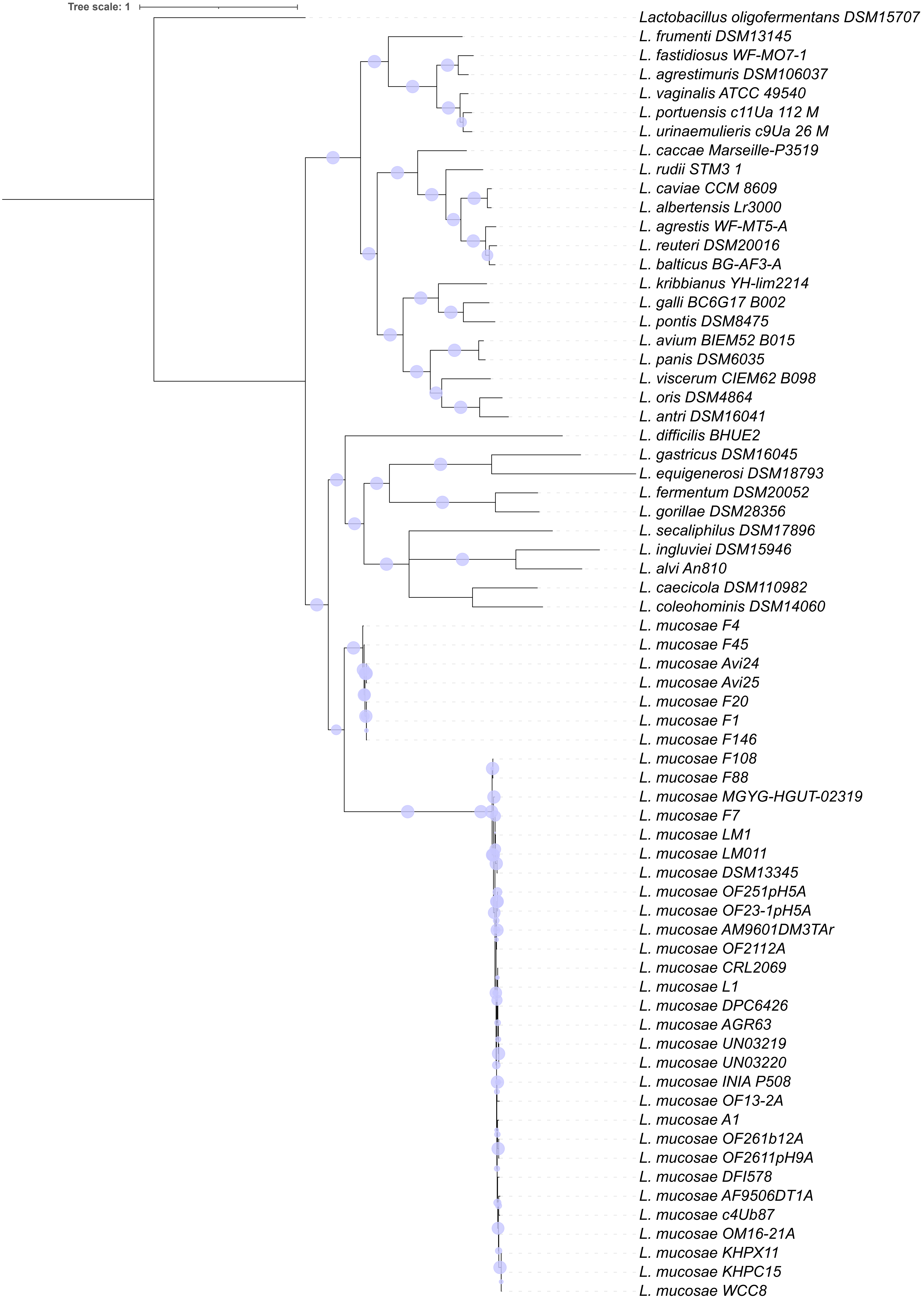

Tree scale: 1
Lactobacillus oligofermentans DSM15707
L. frumenti DSM13145
L. fastidiosus WF-MO7-1
L. agrestimuris DSM106037
L. vaginalis ATCC 49540
L. portuensis c11Ua 112 M
L. urinaemulieris c9Ua 26 M
L. caccae Marseille-P3519
L. rudii STM3 1
L. caviae CCM 8609
L. albertensis Lr3000
L. agrestis WF-MT5-A
L. reuteri DSM20016
L. balticus BG-AF3-A
L. kribbianus YH-lim2214
L. galli BC6G17 B002
L. pontis DSM8475
L. avium BIEM52 B015
L. panis DSM6035
L. viscerum CIEM62 B098
L. oris DSM4864
L. antri DSM16041
L. difficilis BHUE2
L. gastricus DSM16045
L. equigenerosi DSM18793
L. fermentum DSM20052
L. gorillae DSM28356
L. secaliphilus DSM17896
L. ingluviei DSM15946
L. alvi An810
L. caecicola DSM110982
L. coleohominis DSM14060
L. mucosae F4
L. mucosae F45
L. mucosae Avi24
L. mucosae Avi25
L. mucosae F20
L. mucosae F1
L. mucosae F146
L. mucosae F108
L. mucosae F88
L. mucosae MGYG-HGUT-02319
L. mucosae F7
L. mucosae LM1
L. mucosae LM011
L. mucosae DSM13345
L. mucosae OF251pH5A
L. mucosae OF23-1pH5A
L. mucosae AM9601DM3TAr
L. mucosae OF2112A
L. mucosae CRL2069
L. mucosae L1
L. mucosae DPC6426
L. mucosae AGR63
L. mucosae UN03219
L. mucosae UN03220
L. mucosae INIA P508
L. mucosae OF13-2A
L. mucosae A1
L. mucosae OF261b12A
L. mucosae OF2611pH9A
L. mucosae DFI578
L. mucosae AF9506DT1A
L. mucosae c4Ub87
L. mucosae OM16-21A
L. mucosae KHPX11
L. mucosae KHPC15
L. mucosae WCC8
